# Impaired humoral and T helper cell responses to toxic shock syndrome toxin-1 (TSST-1) in granulomatosis with polyangiitis

**DOI:** 10.1093/rheumatology/keaf439

**Published:** 2025-08-18

**Authors:** Hang Liao, Abraham Rutgers, Minke G Huitema, Caroline Roozendaal, Peter Heeringa, Wayel H Abdulahad

**Affiliations:** Department of Pathology and Medical Biology, University Medical Center Groningen, University of Groningen, Groningen, The Netherlands; Department of Rheumatology and Clinical Immunology, University Medical Center Groningen, University of Groningen, Groningen, The Netherlands; Department of Rheumatology and Clinical Immunology, University Medical Center Groningen, University of Groningen, Groningen, The Netherlands; Department of Laboratory Medicine, University Medical Center Groningen, University of Groningen, Groningen, The Netherlands; Department of Pathology and Medical Biology, University Medical Center Groningen, University of Groningen, Groningen, The Netherlands; Department of Pathology and Medical Biology, University Medical Center Groningen, University of Groningen, Groningen, The Netherlands; Department of Rheumatology and Clinical Immunology, University Medical Center Groningen, University of Groningen, Groningen, The Netherlands

**Keywords:** granulomatosis with polyangiitis, T cells, ANCA, TSST-1, peripheral helper T cell, TCR-Vβ2, IL-21

## Abstract

**Objectives:**

Nasal carriage of toxic shock syndrome toxin-1 (TSST-1)-positive *Staphylococcus aureus* (SA) is associated with a high relapse rate in granulomatosis with polyangiitis (GPA), suggesting TSST-1’s role in disease progression. This study investigated the immune response to *S. aureus*-derived TSST-1 and characterized TSST-1-specific Th cells in GPA patients.

**Methods:**

Plasma anti-TSST-1 IgG levels were measured in 45 remission GPA patients and 25 healthy controls (HCs) using ELISA. Circulating TSST-1-reactive CD4^+^T helper (Vβ2^+^Th) cells were analysed and sub-classified in 62 remission GPA patients and 22 HCs by flow cytometry. Additionally, PBMCs were stimulated *in vitro* with TSST-1 for 14 days to evaluate its effect on PR3-ANCA production and CD4^+^Th cell cytokine production (IFNγ, IL-4, IL-17, IL-21) measured by Phadia ImmunoCAP^®^250 and flow cytometry, respectively.

**Results:**

GPA patients with nasal SA carriage (SA+) had lower plasma anti-TSST-1 IgG levels and reduced numbers of circulating Vβ2^+^Th cells compared with HCs. An increased frequency of Vβ2^+^Th cells expressing the phenotype of peripheral helper T cells (T_P_h; PD-1^High^CXCR5^Neg^) was observed in SA+ GPA patients compared with HCs. TSST-1 stimulation enhanced IL-21 production in Th cells from SA+ patients and induced PR3-ANCA production *in vitro* in a subset of GPA patients. Notably, numbers of circulating Vβ2^+^Th cells correlated positively with increased relapse-free survival in SA+ GPA patients.

**Conclusion:**

Impaired humoral immunity against Vβ2-restricted superantigens in SA+ GPA patients may lead to insufficient clearance of TSST-1. TSST-1-driven IL-21 production could enhance ANCA production and promote disease progression. Reduced Vβ2^+^Th cells may increase relapse risk, emphasizing their potential value as a tool for monitoring disease progression.

## Graphical abstract

**Figure:**
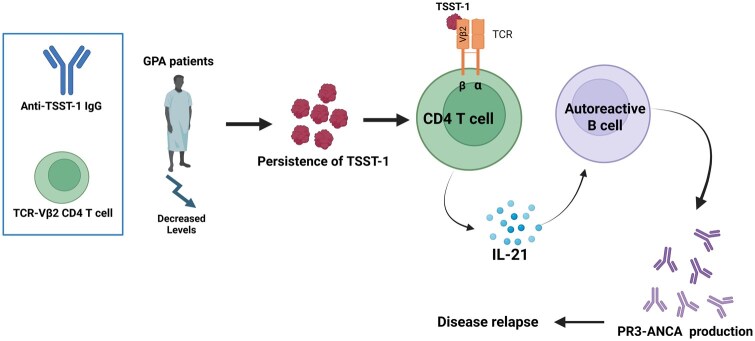

Rheumatology key messages

- Reduced plasma anti-TSST-1 IgG levels and Vβ2^+^Th cell numbers in SA^+^GPA patients suggest insufficient humoral and cellular immunity for TSST-1 clearance.
- TSST-1 potentiates IL-21 production by Th cells in SA^+^GPA patients and enhances PR3-ANCA production *in vitro*.
- Increased numbers of circulating Vβ2^+^Th cells are associated with a reduced risk of relapse in SA^+^GPA patients.

## Introduction

Granulomatosis with polyangiitis (GPA) is a systemic autoimmune disease that affects small- to medium-sized blood vessels, primarily causing severe respiratory tract or kidney damage [1]. GPA is characterized by the presence of ANCAs targeting PR3 [2]. Patients with GPA are prone to disease relapse, leading to progressive organ function loss and increased comorbidities. Various genetic and environmental factors contribute to the inflammatory process in GPA, but the primary cause remains unknown [3]. Clinical observations indicate that 60–70% of GPA patients are chronic nasal carriers of *Staphylococcus aureus* (*S. aureus*), which significantly increases the risk of relapse [4–7]. As part of maintenance therapy, antibacterial treatments such as cotrimoxazole can reduce the risk of relapse [8]. These findings suggest that *S. aureus* plays an essential role in GPA pathogenesis.

*Staphylococcus aureus*, a common gram-positive bacterium, is frequently found in the anterior nares of the nose and is usually asymptomatic [9–12]. However, it can act as an opportunistic pathogen, causing diseases ranging from skin infections to life-threatening pneumonia and sepsis [13]. The pathogenicity of *S. aureus* is largely due to its virulence factors, including superantigens (SAgs), such as toxic shock syndrome toxin-1 (TSST-1) and staphylococcal enterotoxins (SEA, SEB). These SAgs can potently induce T cell proliferation and cytokine production by cross-linking the variable regions of particular T cell receptor β chains (TCR-Vβ) with MHC class II molecules on antigen-presenting cells outside the antigen-binding groove [14]. Additionally, *S. aureus* activates T cells through factors such as α-toxin and cell wall components, such as peptidoglycan (PGN) and lipoteichoic acid (LTA), contributing to the pathogenic process. Therefore, it can be suggested that the virulence factors of *S. aureus* trigger T cell activation and contribute to the pathological mechanisms underlying GPA.

In GPA, we previously demonstrated a skewing in circulating CD4^+^Th cell subsets towards the pro-inflammatory effector memory phenotype [15, 16]. In particular, IL-21-producing CD4^+^T follicular helper cells (T_F_h) and IL-17-producing Th cells (Th_17_) are significantly increased in GPA patients [17, 18]. Importantly, we have also shown that GPA patients carrying TSST-1 have an increased risk of disease relapse [6]. The link between TSST-1 positive *S. aureus* carriage (SA+) and increased relapse rate suggests that TSST-1 may be a driving force for T and B cell activation in GPA. However, evidence at the cellular and molecular level for this contention is lacking. This study aimed to evaluate the humoral immune response to *S. aureus*-derived TSST-1 and to characterize TSST-1-specific Th cells in GPA patients, thereby advancing our understanding of the role of TSST-1 in GPA pathogenesis.

## Methods

### Study population

In total, 62 patients diagnosed with GPA and 47 age- and sex-matched HCs were included. GPA diagnosis was based on the Chapel Hill Consensus Conference definitions [1]. At the time of sampling, all patients were in clinical remission, as defined by the (BVAS of zero and had not received rituximab treatment before. None of the GPA patients or controls had any clinically active infectious disease at blood withdrawal. Clinical and laboratory data are summarized in Supplementary Table S1.

All participants provided informed consent. The study was approved by the UMCG’s medical ethics committee (METc No. 2010.057, 2012/151 and ABR: NL37805.042.12).

### Detection of *S. aureus*

*Staphylococcus aureus* carriage was determined by swabbing both anterior nares. Swabs were cultured on 5% sheep blood and mannitol salt agar for 72 h at 37°C. *Staphylococcus aureus* was identified by coagulase and DNase positivity. Chronic carriage was defined when ≥50% of cultures showed *positivity*.

### Sample preparation

Peripheral blood mononuclear cells (PBMCs) were isolated using density-gradient centrifugation methods, frozen and thawed as described [19]. Plasma samples were collected from EDTA-anticoagulated blood and stored at −20°C.

### Fluorescent cell barcoding

PBMCs were stimulated *in vitro* with staphylococcal superantigens and bacterial components, and intracellular cytokines in CD4^+^ Th cells were determined using a fluorescent cell barcoding (FCB) assay, as detailed in Supplementary Data S1 [20].

### ELISA

Plasma concentrations of anti-TSST-1 IgG were determined using the Human Toxic Shock Syndrome Toxin 1 Antibody ELISA Kit (Catalogue#: MBS109562, Mybiosource, San Diego, USA) according to the manufacturer’s instructions. The detection range was 0.25–8 ng/ml and sensitivity was 0.1 ng/ml. Concentrations lower than the minimum detection range are shown as half of the minimum detection value (0.125 ng/ml).

Levels of anti-SEA and anti-SEB IgG antibodies in the plasma samples were measured by an in-house indirect ELISA (Supplementary Data S2). Plasma levels of anti-SpeC IgG were determined according to a previously published protocol [21] (Supplementary Data S2).

### Phenotyping of TSST-1 specific (Vβ2^+^) Th cells

After thawing, PBMCs were washed and incubated with Zombie UV™ Fixable Viability Kit (BioLegend, San Diego, USA), anti-human TCR-Vβ2, CD3, CD4, CD45RO, CCR7, PD-1, CXCR5, CD25, CCR6, CXCR3 and CCR4 (details are provided in Supplementary Table S2). Samples were fixed, washed and measured on a BD Symphony A5. Fluorescence minus one (FMO) controls were used to set the gates. Data were analysed using Kaluza software V2.1 (Beckman Coulter, Indianapolis, USA). Using the lymphocyte count, we calculated the absolute numbers of circulating Vβ2^+^Th cells.

### Cell stimulation and quantification of *in vitro* production of IgG and PR3-ANCA

PBMCs were cultured at 1 × 10^6^ cells/ml in polypropylene tubes at 37°C with 5% CO_2_ for 14 days in the presence of 10 pg/ml TSST-1 (Sigma-Aldrich, Darmstadt, Germany) and 20 μg/ml BAFF (PeproTech Inc., Rocky Hill, USA). Negative controls were treated with only BAFF, while positive controls were stimulated with 3.2 μg/ml CpG-ODN 2006 (Hycult Biotech, Uden, The Netherlands), 20 μg/ml BAFF and 10 μg/ml IL-21 (Immunotools, Friesoythe, Germany). After 14 days, supernatants were collected to measure PR3-ANCA and total IgG levels. PR3-ANCA concentrations were measured with a Phadia ImmunoCAP 250 analyser using EliA PR3^S^ (Thermo Fisher Scientific, Waltham, MA, USA), and total IgG was measured with anin-house sandwich ELISA [22], as detailed in Supplementary Data S3.

### Statistical analysis

A Mann–Whitney *U* test was performed between two groups only if the Kruskal–Wallis test showed significant differences among non-paired groups (GPA, HC, SA+, SA−, _NC_SA+ and _NC_SA−). Differences in IgG and PR3-ANCA production were analysed by Wilcoxon signed-rank test. A Mental–Cox test was performed to compare groups in the Kaplan–Meier analysis of relapse-free survival. Statistical significance was set at *P* < 0.05. Data were analysed using GraphPad Prism V10.1 (GraphPad Software, San Diego, USA).

## Results

### Reduced plasma levels of anti-TSST-1 in SA+ GPA patients

To investigate whether GPA patients in remission have an effective humoral immune response against TSST-1, we measured the IgG levels of anti-TSST-1, anti-SEA, anti-SEB and total IgG in the plasma of SA+ and SA− GPA patients in remission, as well as in age- and sex-matched HCs.

Patients who receive cotrimoxazole treatment can yield false-negative results in nasal swab tests for *S. aureus*. Consequently, we divided GPA patients into two groups based on two different criteria. First, all GPA patients were divided into two groups based on *S. aureus* carriership: SA+ (*S. aureus* carrier) and SA− (*S. aureus* non-carrier). Second, excluding those who received cotrimoxazole, GPA patients were then categorized as non-cotrimoxazole SA+ (_NC_SA+) and non-cotrimoxazole SA− (_NC_SA−). All figures follow this categorization unless otherwise specified in the legend.

**Figure 1.**
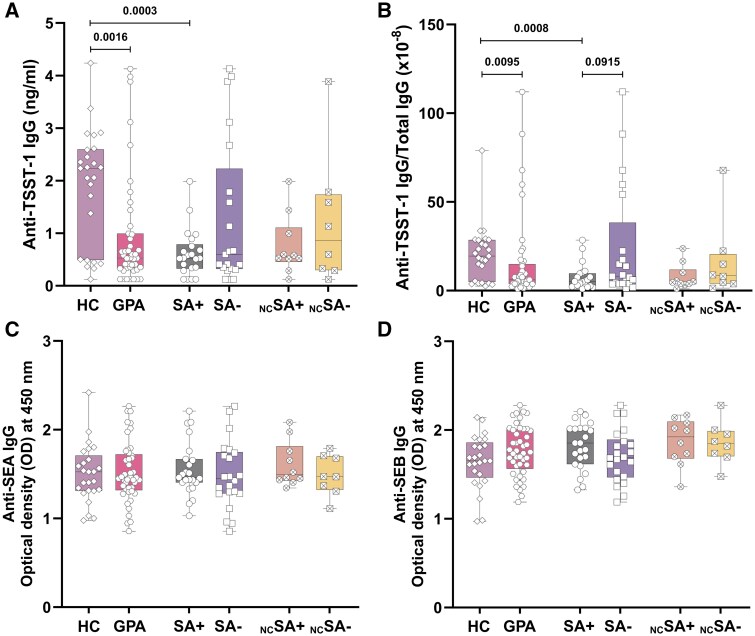
Lower anti-TSST-1 IgG levels in plasma of SA+ GPA patients compared with HCs. Concentration of anti-TSST-1 IgG (ng/ml) (**A**), anti-SEA IgG (OD value) (**C**) and anti-SEB IgG (OD value) (**D**) in plasma samples of GPA patients in remission (*n* = 62). Patients were grouped as SA+ (*n *= 23), SA− (*n *= 22), _NC_SA+ (*n *= 10), _NC_SA− (*n *= 8) and HCs (*n *= 25); (**B**) Ratio of anti-TSST-1 IgG (ng/ml) concentration to total IgG (ng/ml) concentration in plasma of remission GPA patients, same subgroups and HCs; graphs represent Box and Whiskers plots (min to max, median). TSST-1: toxic shock syndrome toxin-1; SA+: *S. aureus* nasal carrier; SA−: *S. aureus* non-carrier; _NC_SA+: non-cotrimoxazole SA*+*; _NC_SA−: non-cotrimoxazole SA−; HC: healthy control

As shown in Fig. 1A, anti-TSST-1 IgG concentrations were significantly lower in SA+ GPA patients than in HCs, regardless of whether they were receiving cotrimoxazole. In contrast, no significant differences were observed between SA− GPA patients and either SA+ GPA patients or HCs. To account for variations in total IgG levels among patients, we calculated the ratio of anti-TSST-1 IgG to total IgG. This analysis revealed a significant reduction in the anti-TSST-1 IgG/total IgG ratio in SA+ GPA patients compared with HCs (Fig. 1B).

In contrast, plasma levels of anti-SEA IgG and anti-SEB IgG in GPA patients were comparable to those in HCs (Fig. 1C and D).

Since TSST-1, unlike SEA and SEB, is known to signal exclusively through the Vβ2 chain on Th cells, we next assessed the humoral immune response to SpeC (Streptococcal pyrogenic exotoxin C), another superantigen that also signal through Vβ2 chain [14]. Interestingly, we observed a similar reduction in plasma levels of anti-SpeC IgG in GPA patients compared with HCs (Supplementary Fig. S2).

Collectively, these results suggest that GPA patients, particularly SA+ GPA patients, exhibit an inadequate humoral immune response to superantigens that signal exclusively through the Vβ2 chain. Notably, the impaired response to TSST-1 is of particular relevance, as persistent *S. aureus* carriage is a well-established risk factor for relapse in GPA patients [4, 6].

### Decreased numbers of circulating Vβ2^+^Th cells in GPA patients

**Figure 2.**
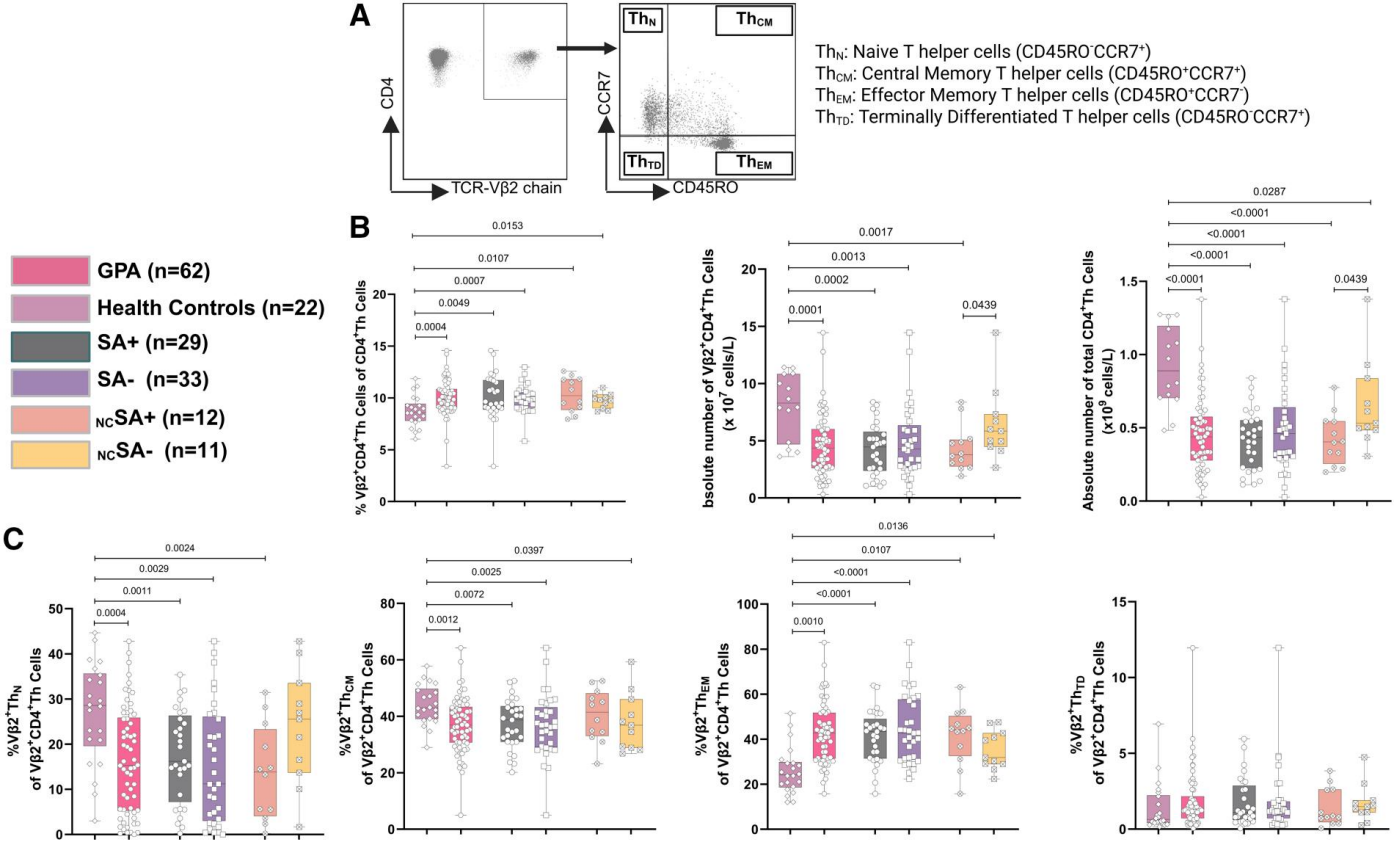
Absolute numbers and frequencies of circulating TCR-Vβ2^+^CD4^+^Th cells in GPA patients. (**A**) Representative flow cytometry dot plots showing TSST-1-reactive TCR-Vβ2^+^CD4^+^Th cells (left) and their subsets based on CD45RO/CCR7 expression (right) in a GPA patient in remission. (**B**) Percentage of TCR-Vβ2^+^CD4^+^Th cells among total CD4^+^Th cells (left), absolute TCR-Vβ2^+^CD4^+^Th cell counts (middle) and total CD4^+^Th cell counts (right). Lymphocyte counts were unavailable for eight healthy controls because of incomplete records. (**C**) Percentage of Vβ2^+^Th_N_, Vβ2^+^Th_CM_, Vβ2^+^Th_EM_ and Vβ2^+^Th_TD_ cells among Vβ2^+^CD4^+^Th cells in GPA patients and healthy controls. Graphs represent Box and Whiskers plots (min to max, median); SA+: *S. aureus* nasal carrier; SA−: *S. aureus* non-carrier; _NC_SA+: non-cotrimoxazole SA*+*; _NC_SA−: non-cotrimoxazole SA*−*

To examine the impact of TSST-1 on circulating CD4^+^ helper T (Th) cells, we first evaluated the frequency of circulating TSST-1-reactive Th cells, identified as CD3^+^CD4^+^TCR-Vβ2^+^ cells (Vβ2^+^Th cells) (Fig. 2A). As shown in Fig. 2B, the frequency of Vβ2^+^Th cells was significantly increased in all GPA patients compared with HCs. However, no significant differences were observed between SA+ and SA− GPA patients.

Next, we assessed the absolute count of circulating Vβ2^+^Th cells. Unlike the frequency, the absolute numbers of Vβ2^+^Th cells were significantly decreased in both SA+ and SA− GPA patients compared with HCs, regardless of whether they received cotrimoxazole treatment (Fig. 2B). The reduction in Vβ2^+^Th cell numbers in GPA patients relative to HCs was attributed to a decrease in the total number of CD4^+^T cells, as illustrated in Fig. 2B. Since maintenance therapy with AZA is known to reduce lymphocyte counts, we investigated whether AZA treatment was associated with changes in Vβ2^+^Th cell numbers. However, no such correlation was observed (Supplementary Fig. S3). Interestingly, _NC_SA+ patients exhibited lower numbers of circulating Vβ2^+^Th cells compared with _NC_SA− patients (Fig. 2B). These results suggest that, although Th cells from GPA patients appear to respond to TSST-1, as indicated by an increased proportion of Vβ2^+^Th cells, the observed reduction in their absolute count in the peripheral blood, particularly in _NC_SA+ patients, may reflect insufficient T cell immunity for the effective clearance of TSST-1 in these patients.

### Phenotypic skewing of circulating TSST-1 reactive Vβ2^+^Th cells in GPA patients from naïve to effector memory

We next evaluated the differences in the distribution of naïve and memory Vβ2^+^Th cell subsets in SA+ and SA− GPA patients and HCs. We found that, compared with HCs, both SA+ and SA− GPA patients had decreased frequencies of Th_N_ and Th_CM_ cells but an increased frequency of Th_EM_ within the Vβ2^+^Th cell population, while no significant change was observed in the frequency of Vβ2^+^Th_TD_ cells (Fig. 2C). These results suggest that Vβ2^+^Th cells in GPA patients are continuously activated, as evidenced by the decreased Th_N_ and increased Vβ2^+^Th_EM_ cell proportions, which typically require consistent and strong stimulation to develop.

### Distribution of TSST-1 reactive Vβ2^+^ memory Th cell subset in SA+ and SA− GPA patients

**Figure 3.**
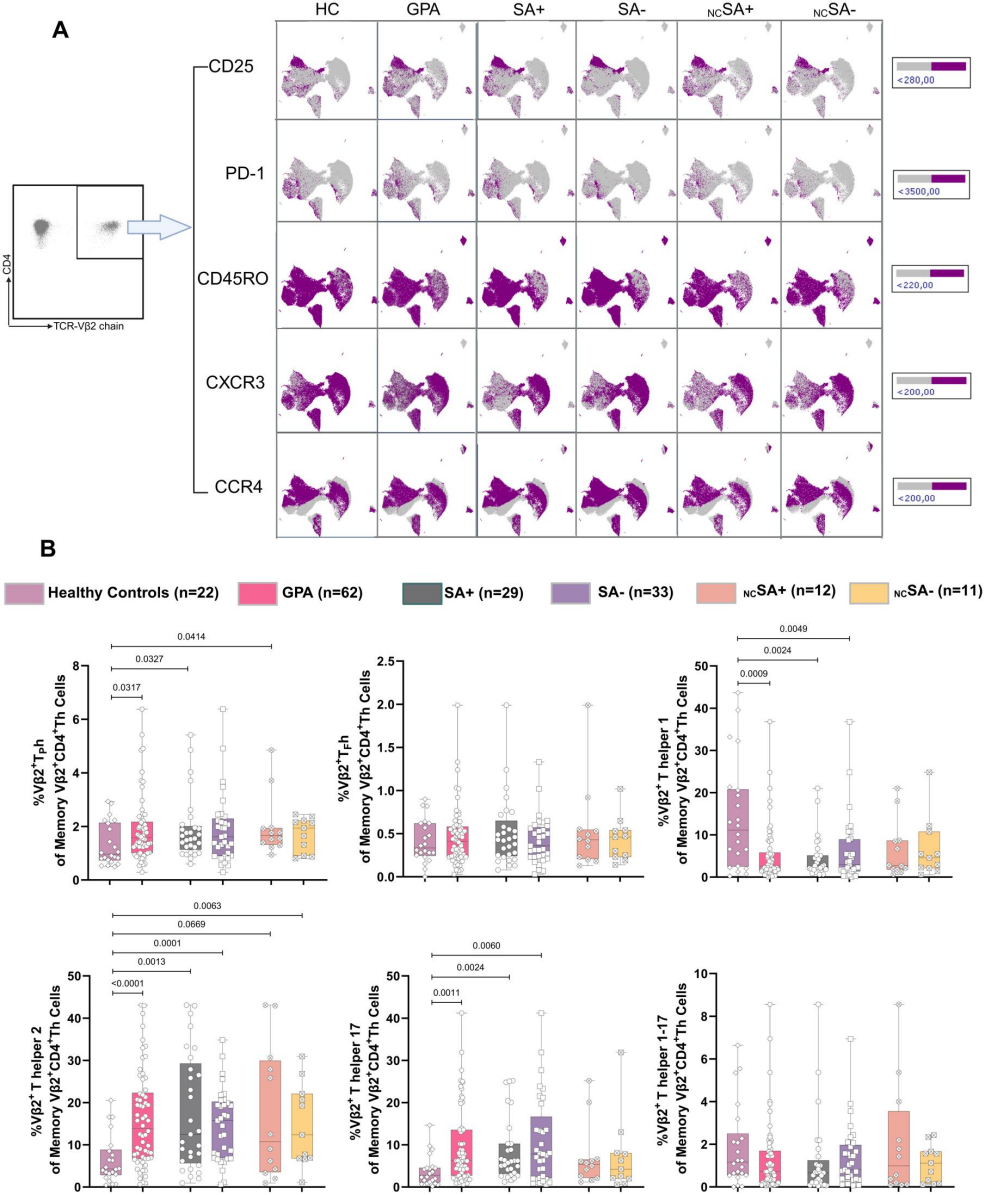
Distinct phenotypes of circulating Vβ2^+^CD4^+^Th cells in GPA patients. (**A**) UMAP visualization of five single-marker expressions in Vβ2^+^Th cells, based on 3000 cells per sample from merged samples from GPA patients, and HCs. Thresholds were defined by fluorescence intensity used in manual gating with Kaluza. (**B**) Percentages of memory T_P_h, T_F_h, Th_1_, Th_2_, Th_17_ and Th_1–17_ within the total memory Vβ2^+^Th cells in GPA patients and HCs. Graphs represent Box and Whiskers plots (min to max, median). T_P_h: T peripheral helper cell; T_F_h: T follicular helper cell; SA+: *S. aureus* nasal carrier; SA−: *S. aureus* non-carrier; _NC_SA+: non-cotrimoxazole SA*+*; _NC_SA−: non-cotrimoxazole SA*−*

We next conducted a detailed phenotypic characterization of memory Vβ2^+^Th cells. UMAP analysis revealed notable differences in PD-1, CD45RO, CXCR3 and CCR4 expression on Vβ2^+^Th cells among SA+, SA− and HCs (Fig. 3A and Supplementary Fig. S4A). To explore this further, we zoomed in on the distinct phenotypic distribution of the Vβ2^+^ memory Th cell subsets by analysing their expression of function-related surface markers (Supplementary Fig. S4B).

Accordingly, TSST-1-reactive Vβ2^+^ memory Th cells were sub-classified into Th_1_, Th_2_, Th_17_, Th_1–17_, T_F_h and T_P_h cell subsets (Supplementary Fig. S4B). Compared with HCs, SA+ GPA patients, including _NC_SA+ GPA patients, exhibited an increased frequency of the memory Vβ2^+^T_p_h cell subset (Fig. 3B). Both SA+ and SA− GPA patients consistently showed higher frequencies of Vβ2^+^Th_2_ and Vβ2^+^Th_17_ memory subsets and a lower frequency of the Vβ2^+^Th_1_ memory cell subset than HCs (Fig. 3B). No differences were observed in Vβ2^+^T_F_h, Vβ2^+^Th_1–17_ cell and Vβ2^+^ memory Treg cell subsets (Fig. 3B and Supplementary Fig. S4C).

These results suggest that TSST-1 reactive Vβ2^+^memory Th cells from SA+ GPA patients express a pro-inflammatory phenotype, which may contribute to disease pathogenesis.

### TSST-1 potentiates IL-21 production by Th cells in SA+ GPA patients

**Figure 4.**
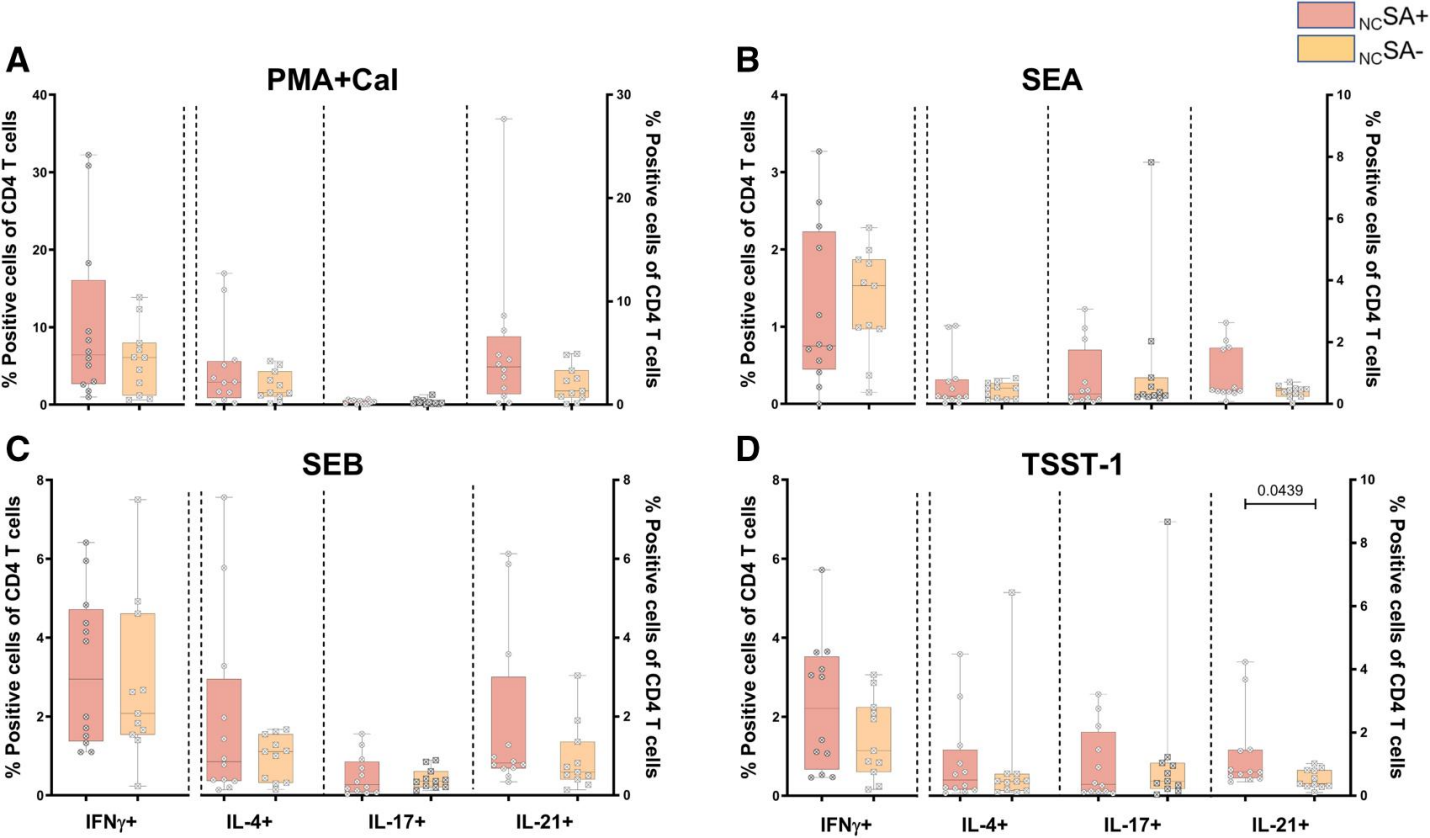
TSST-1 induces a higher percentage of IL-21 producing CD4^+^Th cells in _NC_SA+ GPA patients compared with _NC_SA− GPA patients. Data are presented as percentages of IFNγ^+^, IL-4^+^, IL-17^+^ and IL-21^+^ cells within the total CD4^+^Th cell population, in _NC_SA+ (*n* = 12) and _NC_SA− (*n* = 11) GPA patients, after *in vitro* stimulation of PBMCs with PMA + Ca-I (**A**), SEA (**B**), SEB (**C**) or TSST-1 (**D**). Graphs represent Box and Whiskers plots (min to max, median). PMA: phorbol 12-myristate 13-acetate; Ca-I: calcium ionophore; SEA: Staphylococcal enterotoxin A; SEB: Staphylococcal enterotoxin B; TSST-1: toxic shock syndrome toxin-1; PBMCs: peripheral blood mononuclear cells; _NC_SA+: non-cotrimoxazole *S. aureus* nasal carrier; _NC_SA−: non-cotrimoxazole *S. aureus* non-carrier

To investigate the functional impact of TSST-1 on the Th cells, we assessed the intracellular production of pro-inflammatory cytokines (IFNγ, IL-4, IL-17 and IL-21) in Th cells from _NC_SA+ and _NC_SA− GPA patients following *in vitro* stimulation with either TSST-1 or other SA super antigens, and control stimulation (PMA and Ca-I) as described in Supplementary Fig. S1A. All stimuli tested enhanced cytokine production in Th cells from both _NC_SA+ and _NC_SA− patients (Fig. 4 and Supplementary Fig. S1B). No significant differences were detected in the overall cytokine patterns of Th cells between _NC_SA+ and _NC_SA− patients. However, among the cytokines evaluated, the only significant difference between _NC_SA+ and _NC_SA− patients was observed in IL-21 production, which was enhanced in _NC_SA+ patients following *in vitro* stimulation with TSST-1 (Fig. 4D).

Overall, these results demonstrate the impact of TSST-1 on Th cell cytokine production in general, specifically promoting a shift towards an IL-21-producing Th cell subset in SA+ patients. This skewing may influence autoantibody production, as IL-21 is a key driver of (auto)antibody production.

### TSST-1 induces higher IgG production and may contribute to PR3-ANCA production *in vitro*

**Figure 5.**
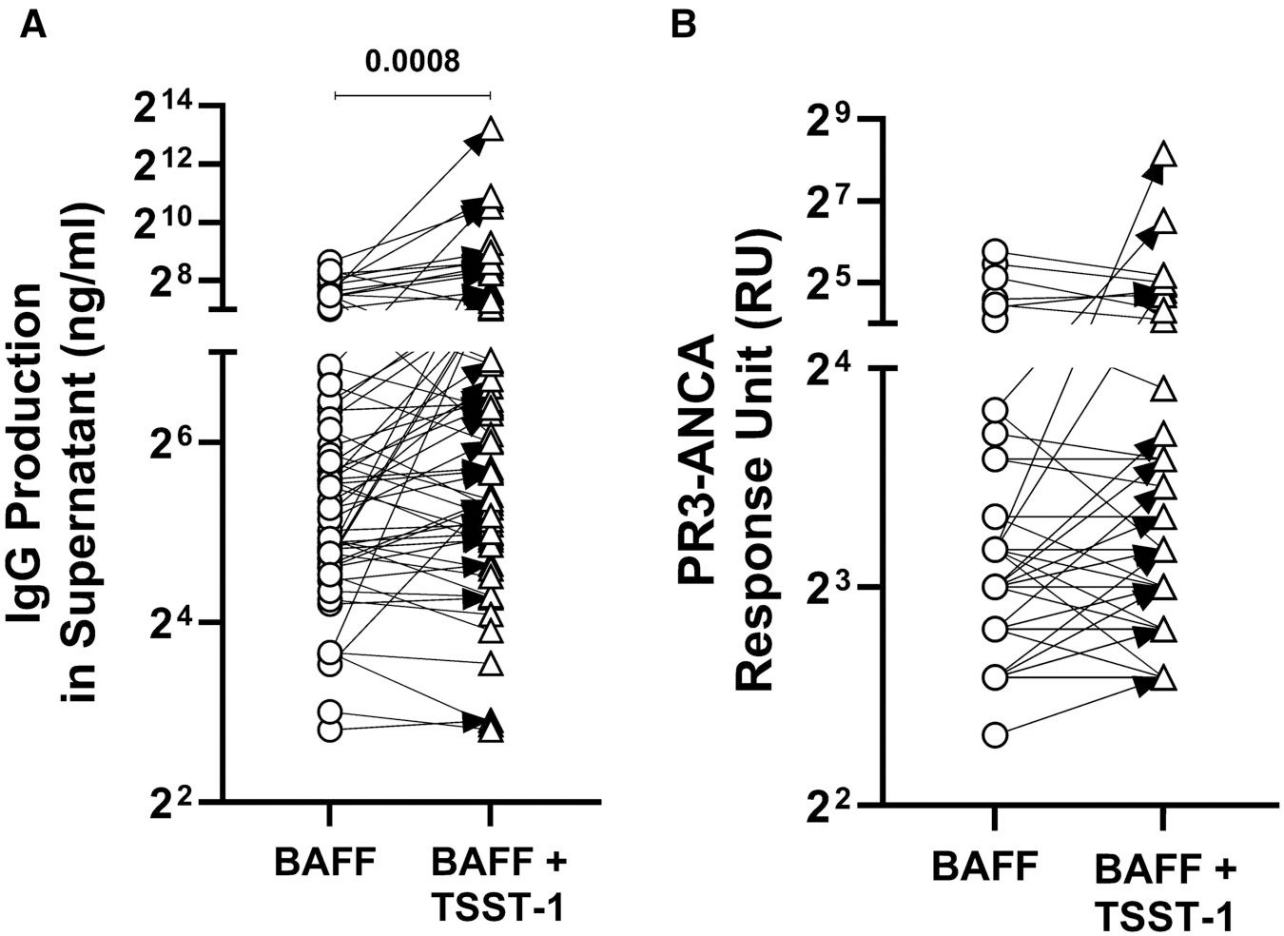
TSST-1 induces IgG and PR3-ANCA production *in vitro*. PBMCs from remission GPA patients (*n* = 62) were cultured in the presence of BAFF with or without TSST-1. Culture supernatants were collected after 14 days to measure total IgG by ELISA (**A**) and PR3-ANCA (**B**) by Phadia ImmunoCAP^®^ 250 Analyser. Circle: BAFF only group; triangle: BAFF plus TSST-1 group. Black arrows represent individual GPA patients (*n* = 24, 38.7%) who produced higher PR3-ANCAs (BAFF plus TSST-1 group minus BAFF only group >0) upon stimulation with TSST-1. The same concentration in different samples result in spot overlapping in the figures. PBMCs: peripheral blood mononuclear cells; BAFF: B cell activating factor; TSST-1: toxic shock syndrome toxin-1

To investigate whether TSST-1 can induce PR3-ANCA production, we stimulated PBMCs from GPA patients with TSST-1 for 14 days. As shown in Fig. 5A, TSST-1 significantly enhanced total IgG production. PR3-ANCA was also enhanced in over one-third of GPA patients (*n* = 22, 38.7%) (Fig. 5B). This suggests that TSST-1 may influence PR3-ANCA production *in vitro*, and could be involved in mechanisms relevant to disease relapse.

### Increased numbers of circulating Vβ2^+^Th cells are associated with a reduced risk of relapse in SA+ GPA patients

The lower numbers of Vβ2^**+**^Th cell in GPA patients, compared with HCs, may lead to an inadequate humoral immune response to TSST-1, impairing the ability to eliminate this superantigen. The persistence of TSST-1 may subsequently contribute to disease progression and relapse. Therefore, tracking changes in circulating Vβ2^**+**^Th cell numbers in GPA patients may be a valuable tool for monitoring disease progression.

**Figure 6.**
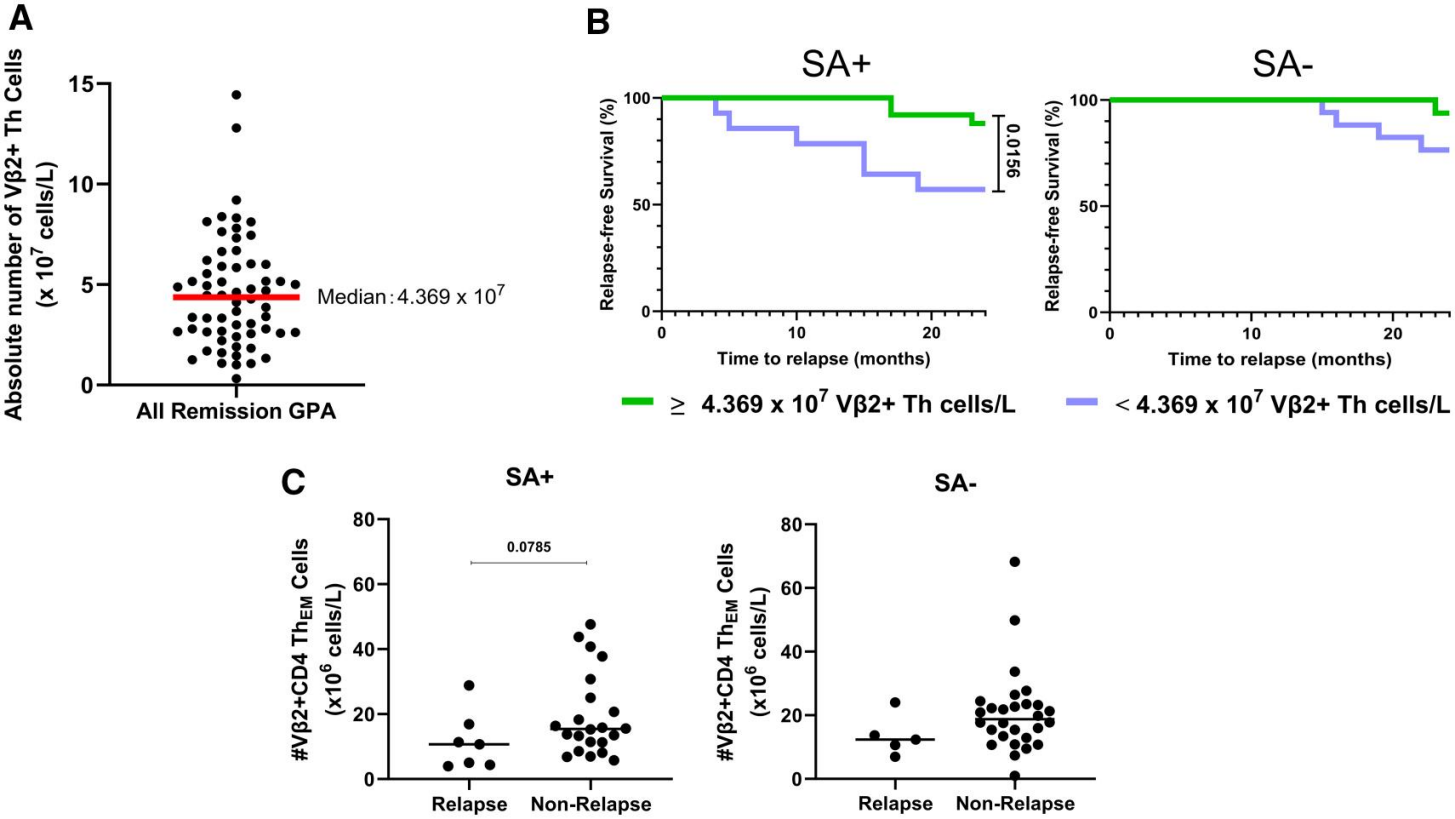
Association between the absolute number of circulating Vβ2^+^Th cells and risk of disease relapse. (**A**) The median number of Vβ2^+^Th cells in all remission GPA patients. (**B**) The Kaplan–Meier curve of the probability of relapse-free survival in SA+ (*n* = 29) and SA− (*n* = 33) GPA patients in remission with lower or higher than 4.369 × 10^7^ cell/l of circulating TCR-Vβ2^+^CD4^+^Th cells for 24 months. Hazard ratios (Mantel–Haenszel) are 5.82 (left) (95% CI: 1.399–24.82) and 3.572 (right) (95% CI: 0.6170–20.68). (**C**) Absolute number of Vβ2^+^CD4 Th_EM_ in patients who relapsed or did not relapse in the following 24 months within SA+ and SA− GPA patients separately. Line represent Median. SA+: *S. aureus* nasal carrier; SA−: *S. aureus* non-carrier.

To evaluate whether the Vβ2^+^Th cell count can serve as a predictor of disease relapse, we investigated whether the absolute number of Vβ2^**+**^Th cells is associated with the risk of future disease relapse. This relationship was analysed separately for SA+ and SA− GPA patients. The patients were divided into two subgroups based on the median Vβ2^**+**^Th cell count across all GPA patients (Fig. 6A): one subgroup included those with fewer than 4.369 × 10^7^ Vβ2^**+**^Th cells/l, while the other subgroup comprised patients with 4.369 × 10^7^ or more Vβ2^**+**^Th cells/l. We found that SA+ patients with fewer than 4.369 × 10^7^ Vβ2^**+**^Th cells/l had a higher risk of disease relapse in the following 24 months compared with SA+ patients with 4.369 × 10^7^ or more Vβ2^**+**^Th cells/l (Fig. 6B). Moreover, SA+ patients who experienced disease relapse within the following 24 months showed a tendency towards reduced absolute numbers of Vβ2^**+**^Th_EM_ cells (Fig. 6C). However, this trend was not observed in SA− GPA patients.

## Discussion

Our study demonstrates that SA+ GPA patients exhibit reduced levels of anti-TSST-1 IgG in their plasma. Correspondingly, we observed decreased numbers of circulating TSST-1 reactive Vβ2^+^Th cells, particularly in _NC_SA+ patients. These Vβ2^+^Th cells show a phenotypic shift from Th_N_ and Th_CM_ towards Th_EM_ cells in all GPA patients. Analysis of the function-related phenotype of memory Vβ2^+^Th cells revealed an increased proportion of T_P_h cells in SA+ GPA patients compared with that in HCs. Additionally, TSST-1 was shown to be involved in IL-21 production in Th cells from _NC_SA+ GPA patients, which we hypothesize may originate from the T_P_h subset, as these cells increase in number and are capable of producing IL-21. Furthermore, in some patients, TSST-1 was shown to induce ANCA production *in vitro*. Notably, although based on a relatively small sample size, our data suggest a potential association between higher numbers of circulating Vβ2^+^Th cells and a reduced risk of future relapse in SA+ GPA patients. However, this observation remains preliminary and requires validation in larger, longitudinal cohorts.

Our observation of a diminished humoral immune response to TSST-1 in SA+ GPA patients is consistent with previously reported data by Glasner *et al.* [23]. Interestingly, antibody titres against other *S. aureus* superantigens, including SEA and SEB, were comparable between GPA patients and healthy controls. This pattern suggests a selective, rather than global, impairment in superantigen-specific humoral immunity. It is important to note that GPA patients not receiving RTX typically retain the ability to mount effective antibody responses to unrelated antigens, such as subunit influenza vaccines, adenovirus vector-based SARS-CoV-2 vaccines and mRNA SARS-CoV-2 vaccines, despite ongoing immunosuppressive therapy [24–27]. Given that TSST-1 selectively engages the Vβ2 chain of the T cell receptor on CD4^+^ T cells, we extended our analysis to SpeC, a streptococcal superantigen that also signals via Vβ2. Consistent with the TSST-1 findings, serum levels of anti-SpeC IgG were also reduced in GPA patients, reinforcing the hypothesis of an impairment in humoral immunity directed against Vβ2-restricted superantigens. The observed deficiency in anti-TSST-1 responses may have functional consequences, as insufficient neutralization and clearance of TSST-1 could contribute to persistent immune activation in these patients.

However, patients with Kawasaki disease, another form of autoimmune vasculitis, exhibited elevated anti-SpeC IgG levels of while maintaining anti-TSST-1 IgG titres comparable to those observed in healthy controls [21, 28]. This distinction underscores a disease-specific dysregulation of humoral immunity in GPA, affecting the response to Vβ2-restricted TSST-1. Given that persistent *S. aureus* colonization is a well-established risk factor for GPA relapse, the inadequate antibody response to TSST-1 may carry clinically relevant implications for disease, persistence and recurrence. While our findings are compelling, future studies involving larger, longitudinally followed cohorts are required to validate and expand upon these observations.

By assessing the percentage of circulating TSST-1 reactive Vβ2^+^Th cells among CD4^+^Th cells, we observed that they were higher in GPA patients than in HCs. Consistent with our findings, a previous study by Simpson *et al.* also demonstrated increased Vβ2 gene expression in the T cells of patients with GPA compared with that in HCs [29]. Vβ2^+^Th cells have also been shown to be expanded in Kawasaki disease, which is thought to be associated with the presence of TSST-1^+^  *S. aureus* [30–33]. The observed increase in the percentage of Vβ2^+^Th cells may indicate that Th cells from remission GPA patients retain the potential to respond to TSST-1 stimulation. These findings also align with the observation of ongoing T cell activation in GPA patients, even during disease remission [16, 34].

A detailed phenotypic analysis of memory Vβ2^+^Th cells, by using function-related surface markers, demonstrated that SA+ GPA patients exhibited an increased frequency of the memory Vβ2^+^T_P_h cell subset. T_P_h cells have been shown to assist B cells in inflamed tissues in autoimmune diseases, act as major IL-21 producers and contribute to the pathogenesis of a wide range of autoimmune diseases [35]. Consistent with this finding, TSST-1 stimulation *in vitro* enhanced IL-21 production by Th cells from SA+ patients. The skewing towards IL-21-producing Th cells by TSST-1 may influence ANCA production, as IL-21 is a key driver of (auto)antibody production [17]. Notably, TSST-1 can induce PR3-ANCA *in vitro* in over one-third of GPA patients. These observations suggest a potential role for TSST-1 in ANCA production and raise the hypothesis that it may contribute to disease relapse, which requires further in-depth analysis.

In addition to the role of TSST-1, the numbers of circulating TSST-1 reactive Vβ2^+^Th cells were significantly reduced in GPA patients, particularly in _NC_SA+ patients, compared with _NC_SA− patients. The reduction in Vβ2^+^Th cells was a consequence of the overall decrease in total CD4^+^Th cells. This decrease may be due to the use of immunosuppressive drugs, which are commonly employed to transition patients from the active stage of the disease to remission by suppressing T cell proliferation [36, 37]. Combined with the observed inadequate humoral response to TSST-1, this may indicate that, although Th cells from GPA patients are capable of responding to TSST-1, their overall reduced numbers may hinder an adequate response necessary for effective clearance of TSST-1. Importantly, while acknowledging the limited sample size, we observed an association between increased numbers of circulating Vβ2^+^Th cells and a reduced risk of relapse in SA+ GPA patients, a finding that warrants further investigation. Meanwhile, we also observed a decreased number of Vβ2^+^Th_EM_ cells in SA+ GPA patients who experienced future disease relapse. Thus, a decrease in these Vβ2^+^Th cells may result in an inadequate immune response, impairing the ability to eliminate the superantigen TSST-1. The persistence of TSST-1 may subsequently contribute to disease progression and relapse. However, these associations remain observational and exploratory; further validation in larger, prospective cohorts is required. According to our data, monitoring changes in circulating Vβ2^+^Th cell numbers in SA+ GPA patients could potentially serve as a valuable tool for assessing relapse risk, however, the small sample size of our study necessitates caution in interpretation, and these findings should be confirmed in future studies.

This study has several limitations that should be considered. First, the cross-sectional design of our study limited our ability to analyse the temporal dynamics of Vβ2^+^Th cells or to evaluate the influence of SA status on disease relapse. Second, we did not type *S. aureus* strains which were carried by GPA patients, especially for TSST-1-expressing *S. aureus*. Future studies exploring Th-cell infiltration in nasal biopsies could provide further insights. Third, the direct impact of Vβ2^+^Th cells on B cells and ANCA production was not assessed, and should be addressed in future studies.

In conclusion, our study provides preliminary insights into a potential involvement of TSST-1 in GPA pathogenesis. Patients with GPA exhibit a diminished humoral immune response to Vβ2-restricted superantigens, particularly TSST-1, along with reduced numbers of circulating TSST-1 reactive Vβ2^+^Th cells, which may reflect an inadequate immune response for effective clearance of TSST-1. Additionally, TSST-1 stimulates the production of IL-21, which is hypothesized to originate from the T_P_h subset. This, in turn, may trigger ANCA production and contribute to the progression of the disease. Correspondingly, a reduction in circulating Vβ2^+^Th cells may increase the risk of future relapse in SA+ GPA patients. While these observations suggest potential clinical relevance, including the utility of monitoring Vβ2+Th cells or targeting TSST-1, further research is essential before drawing definitive conclusions.

## Supplementary Material

keaf439_Supplementary_Data

## Data Availability

The data underlying this article will be shared on reasonable request to the corresponding author.

## References

[1] Jennette JC , Falk RJ, Bacon PA et al 2012 revised International Chapel Hill Consensus Conference Nomenclature of Vasculitides. Arthritis Rheum 2013;65:1–11. 23045170 10.1002/art.37715

[2] Jennette JC , Hoidal JR, Falk RJ. Specificity of anti-neutrophil cytoplasmic autoantibodies for proteinase 3. Blood 1990;75:2263–4. 2189509

[3] Kitching AR , Anders H-J, Basu N et al ANCA-associated vasculitis. Nat Rev Dis Primers 2020;6:71. 32855422 10.1038/s41572-020-0204-y

[4] Stegeman CA , Tervaert JW, Sluiter WJ et al Association of chronic nasal carriage of *Staphylococcus aureus* and higher relapse rates in Wegener granulomatosis. Ann Intern Med 1994;120:12–7. 8250451 10.7326/0003-4819-120-1-199401010-00003

[5] Laudien M , Gadola SD, Podschun R et al Nasal carriage of *Staphylococcus aureus* and endonasal activity in Wegener’s granulomatosis as compared to rheumatoid arthritis and chronic Rhinosinusitis with nasal polyps. Clin Exp Rheumatol 2010;28:51–5. 20412703

[6] Popa ER , Stegeman CA, Abdulahad WH et al Staphylococcal toxic-shock-syndrome-toxin-1 as a risk factor for disease relapse in Wegener’s granulomatosis. Rheumatology (Oxford) 2007;46:1029–33. 17409134 10.1093/rheumatology/kem022

[7] Salmela A , Rasmussen N, Tervaert JWC, Jayne DRW, Ekstrand A, European Vasculitis Study G. Chronic nasal *Staphylococcus aureus* carriage identifies a subset of newly diagnosed granulomatosis with polyangiitis patients with high relapse rate. Rheumatology (Oxford) 2017;56:965–72. 28339745 10.1093/rheumatology/kex001

[8] Stegeman CA , Tervaert JW, de Jong PE, Kallenberg CG. Trimethoprim-sulfamethoxazole (co-trimoxazole) for the prevention of relapses of Wegener’s granulomatosis. Dutch Co-Trimoxazole Wegener Study Group. N Engl J Med 1996;335:16–20. 8637536 10.1056/NEJM199607043350103

[9] Williams RE. Healthy carriage of *Staphylococcus aureus*: its prevalence and importance. Bacteriol Rev 1963;27:56–71. 14000926 10.1128/br.27.1.56-71.1963 PMC441169

[10] Kluytmans J , van Belkum A, Verbrugh H. Nasal carriage of *Staphylococcus aureus*: epidemiology, underlying mechanisms, and associated risks. Clin Microbiol Rev 1997;10:505–20. 9227864 10.1128/cmr.10.3.505 PMC172932

[11] Wertheim HF , Melles DC, Vos MC et al The role of nasal carriage in *Staphylococcus aureus* infections. Lancet Infect Dis 2005;5:751–62. 16310147 10.1016/S1473-3099(05)70295-4

[12] Eriksen NH , Espersen F, Rosdahl VT, Jensen K. Carriage of *Staphylococcus aureus* among 104 healthy persons during a 19-month period. Epidemiol Infect 1995;115:51–60. 7641838 10.1017/s0950268800058118 PMC2271555

[13] Cheung GYC , Bae JS, Otto M. Pathogenicity and virulence of *Staphylococcus aureus*. Virulence 2021;12:547–69. 33522395 10.1080/21505594.2021.1878688 PMC7872022

[14] Fraser JD , Proft T. The bacterial superantigen and superantigen-like proteins. Immunol Rev 2008;225:226–43. 18837785 10.1111/j.1600-065X.2008.00681.x

[15] Abdulahad WH , Kallenberg CG, Limburg PC, Stegeman CA. Urinary CD4+ effector memory T cells reflect renal disease activity in antineutrophil cytoplasmic antibody-associated vasculitis. Arthritis Rheum 2009;60:2830–8. 19714581 10.1002/art.24747

[16] Abdulahad WH , van der Geld YM, Stegeman CA, Kallenberg CG. Persistent expansion of CD4+ effector memory T cells in Wegener’s granulomatosis. Kidney Int 2006;70:938–47. 16837919 10.1038/sj.ki.5001670

[17] Abdulahad WH , Lepse N, Stegeman CA et al Increased frequency of circulating IL-21 producing Th-cells in patients with granulomatosis with polyangiitis (GPA). Arthritis Res Ther 2013;15:R70. 23799890 10.1186/ar4247 PMC4060544

[18] Abdulahad WH , Stegeman CA, Limburg PC, Kallenberg CG. Skewed distribution of Th17 lymphocytes in patients with Wegener’s granulomatosis in remission. Arthritis Rheum 2008;58:2196–205. 18576340 10.1002/art.23557

[19] Land J , Abdulahad WH, Sanders JS et al Regulatory and effector B cell cytokine production in patients with relapsing granulomatosis with polyangiitis. Arthritis Res Ther 2016;18:84. 27044386 10.1186/s13075-016-0978-1 PMC4820899

[20] Krutzik PO , Clutter MR, Trejo A, Nolan GP. Fluorescent cell barcoding for multiplex flow cytometry. Curr Protoc Cytom 2011;Chapter 6:6.31.1–15. 10.1002/0471142956.cy0631s55 PMC3036011 21207359

[21] Yoshioka T , Matsutani T, Toyosaki-Maeda T et al Relation of streptococcal pyrogenic exotoxin C as a causative superantigen for Kawasaki disease. Pediatr Res 2003;53:403–10. 12595587 10.1203/01.PDR.0000049668.54870.50

[22] Lepse N , Land J, Rutgers A et al Toll-like receptor 9 activation enhances B cell activating factor and interleukin-21 induced anti-proteinase 3 autoantibody production in vitro. Rheumatology (Oxford) 2016;55:162–72. 26320128 10.1093/rheumatology/kev293

[23] Glasner C , van Timmeren MM, Stobernack T et al Low anti-staphylococcal IgG responses in granulomatosis with polyangiitis patients despite long-term *Staphylococcus aureus* exposure. Sci Rep 2015;5:8188. 25641235 10.1038/srep08188 PMC5389034

[24] Saad CGS , Borba EF, Aikawa NE et al Immunogenicity and safety of the 2009 non-adjuvanted influenza A/H1N1 vaccine in a large cohort of autoimmune rheumatic diseases. Ann Rheum Dis 2011;70:1068–73. 21540203 10.1136/ard.2011.150250

[25] Carruthers JE , Wells J, Gupta A et al Response to vaccination against SARS-CoV-2 in patients with antineutrophil cytoplasmic antibody-associated vasculitis with renal involvement. Front Med (Lausanne) 2021;8:817845. 35127773 10.3389/fmed.2021.817845 PMC8811045

[26] Holvast A , Stegeman CA, Benne CA et al Wegener’s granulomatosis patients show an adequate antibody response to influenza vaccination. Ann Rheum Dis 2009;68:873–8. 18625625 10.1136/ard.2008.092924

[27] Wieske L , van Dam KPJ, Steenhuis M et al; T2B! Immunity against SARS-CoV-2 study group. Humoral responses after second and third SARS-CoV-2 vaccination in patients with immune-mediated inflammatory disorders on immunosuppressants: a cohort study. Lancet Rheumatol 2022;4:e338–50. 35317410 10.1016/S2665-9913(22)00034-0 PMC8930018

[28] Matsubara K , Fukaya T, Miwa K et al Development of serum IgM antibodies against superantigens of *Staphylococcus aureus* and Streptococcus pyogenes in Kawasaki disease. Clin Exp Immunol 2006;143:427–34. 16487241 10.1111/j.1365-2249.2006.03015.x PMC1809617

[29] Simpson IJ , Skinner MA, Geursen A et al Peripheral blood T lymphocytes in systemic vasculitis: increased T cell receptor V beta 2 gene usage in microscopic polyarteritis. Clin Exp Immunol 1995;101:220–6. 7544245 10.1111/j.1365-2249.1995.tb08342.x PMC1553271

[30] Jason J , Montana E, Donald JF et al Kawasaki disease and the T-cell antigen receptor. Hum Immunol 1998;59:29–38. 9544237 10.1016/s0198-8859(97)00233-4

[31] Abe J , Kotzin BL, Jujo K et al Selective expansion of T cells expressing T-cell receptor variable regions V beta 2 and V beta 8 in Kawasaki disease. Proc Natl Acad Sci U S A 1992;89:4066–70. 1315049 10.1073/pnas.89.9.4066 PMC525633

[32] Abe J , Kotzin BL, Meissner C et al Characterization of T cell repertoire changes in acute Kawasaki disease. J Exp Med 1993;177:791–6. 8094737 10.1084/jem.177.3.791 PMC2190929

[33] Leung DY , Meissner HC, Fulton DR et al Toxic shock syndrome toxin-secreting *Staphylococcus aureus* in Kawasaki syndrome. Lancet 1993;342:1385–8. 7901681 10.1016/0140-6736(93)92752-f

[34] Popa ER , Stegeman CA, Bos NA, Kallenberg CG, Tervaert JW. Differential B- and T-cell activation in Wegener’s granulomatosis. J Allergy Clin Immunol 1999;103:885–94. 10329824 10.1016/s0091-6749(99)70434-3

[35] Yoshitomi H , Ueno H. Shared and distinct roles of T peripheral helper and T follicular helper cells in human diseases. Cell Mol Immunol 2021;18:523–7. 32868910 10.1038/s41423-020-00529-z PMC8027819

[36] Giles AJ , Hutchinson M-KND, Sonnemann HM et al Dexamethasone-induced immunosuppression: mechanisms and implications for immunotherapy. J Immunother Cancer 2018;6:51. 29891009 10.1186/s40425-018-0371-5 PMC5996496

[37] Nayak SP , Bagchi B, Roy S. Effects of immunosuppressants on T-cell dynamics: understanding from a generic coarse-grained immune network model. J Biosci 2022;47:37137–49. 10.1007/s12038-022-00312-4 PMC9734612 36503907

[38] van der Geest KSM , Abdulahad WH, Teteloshvili N et al Low-affinity TCR engagement drives IL-2-dependent post-thymic maintenance of naive CD4+ T cells in aged humans. Aging Cell 2015;14:744–53. 26010129 10.1111/acel.12353 PMC4568962

