# Supplementary material for: Impaired humoral and T helper cell responses to toxic shock syndrome toxin-1 (TSST-1) in granulomatosis with polyangiitis
Source: Rheumatology (Oxford). 2025 Aug 18;64(12):6309–18. doi: 10.1093/rheumatology/keaf439 (PMC12671868; doi:10.1093/rheumatology/keaf439)
Supplement: keaf439_Supplementary_Data [file keaf439_supplementary_data.docx]

**Supplementary material**

**Supplementary Table S1**. Clinical and laboratory characteristics of the patients with GPA and healthy controls at the time of blood sampling.

| Characteristics | GPA | Healthy Controls |
| --- | --- | --- |
| Total number, n | 62 | 47 |
| Male, n (%) | 34 (55%) | 21 (44.7%) |
| Age, median(range), years | 57 (25-86) | 60 (31-80) |
| PR3-ANCA Positive/ Negative^1^, n | 40/22 | N/A |
| Localized/Generalized, n | 20/42 | N/A |
| Kidney involvement, n | 34 | N/A |
| eGFR^2^, ml/min/1.73 m^2^, median (range) | 68 (17-123) | N/A |
| Cyclophosphamide used in the induction therapy^3^ | 59 | N/A |
| Non/maintenance immunosuppressive therapy^4^, n | 42/20 | N/A |
| *S. aureus* nasal carrier (SA+)/ non-carrier (SA-), n | 29/33 | N/A |
| Non Co-trimoxazole treated SA+/SA-, n | 12/11 | N/A |
| Disease duration, years, median (range) | 9 (0-23) | N/A |
| Number of patients who relapse in the following 24 months^5^, n | 12 | N/A |
| Total previous relapse times median (range) | 1.5 (1-5) | N/A |

^1^ ANCA-positive titer ≥1:40, ANCA-negative＜1:20. ^2^ Estimated Glomerular Filtration Rate (eGFR) calculated using the Chronic Kidney Disease Epidemiology Collaboration (CKD-EPI) equation. ^3^ The remaining three patients did not receive cyclophosphamide during induction therapy of which two were treated with cotrimoxazole alone due to limited ENT (Ear, Nose, and Throat) involvement and one with methotrexate. ^4^ Immunosuppressive maintenance therapy: only Azathioprine (n=9), Azathioprine + prednisolone (n=7), or only prednisolone (n=4). ^5^ Among the relapse patients, five exhibited ENT and lung involvement, two had ENT and joint involvement, one had ENT, lung, and joint involvement, one had lung and joint involvement, one presented with ENT, subglottic, and endobronchial involvement, one had ENT, kidney, and joint involvement, and one showed scleral, kidney, and joint involvement.

**Supplementary Table S2.** List of fluorescence-conjugated antibodies used in flow cytometry analysis.

| **Antigen** | **Clone** | **Conjugation** | **Company and Region** |
| --- | --- | --- | --- |
| TCR-Vβ2 | REA654 | PE | Miltenyi Biotec, Bergisch Gladbach, Germany |
| CD3 | SK7 | BV605 | BD Biosciences, Franklin Lakes, USA |
| CD4 | OKT4 | BV421 | Biolegend, San Diego, USA |
| CD45RO | UCHL1 | BUV737 | BD Biosciences, Franklin Lakes, USA |
| CCR7 | 3D12 | PE-Cy7 | BD Biosciences, Franklin Lakes, USA |
| PD-1 | EH12 | BV786 | BD Biosciences, Franklin Lakes, USA |
| CXCR5 | RF8B2 | BB515 | BD Biosciences, Franklin Lakes, USA |
| CD25 | M-A251 | AF647 | Biolegend, San Diego, USA |
| CCR6 | 11A9 | BV480 | BD Biosciences, Franklin Lakes, USA |
| CXCR3 | G025H7 | APC-Cy7 | Biolegend, San Diego, USA |
| CCR4 | L291H4 | PerCP-Cy5.5 | Biolegend, San Diego, USA |


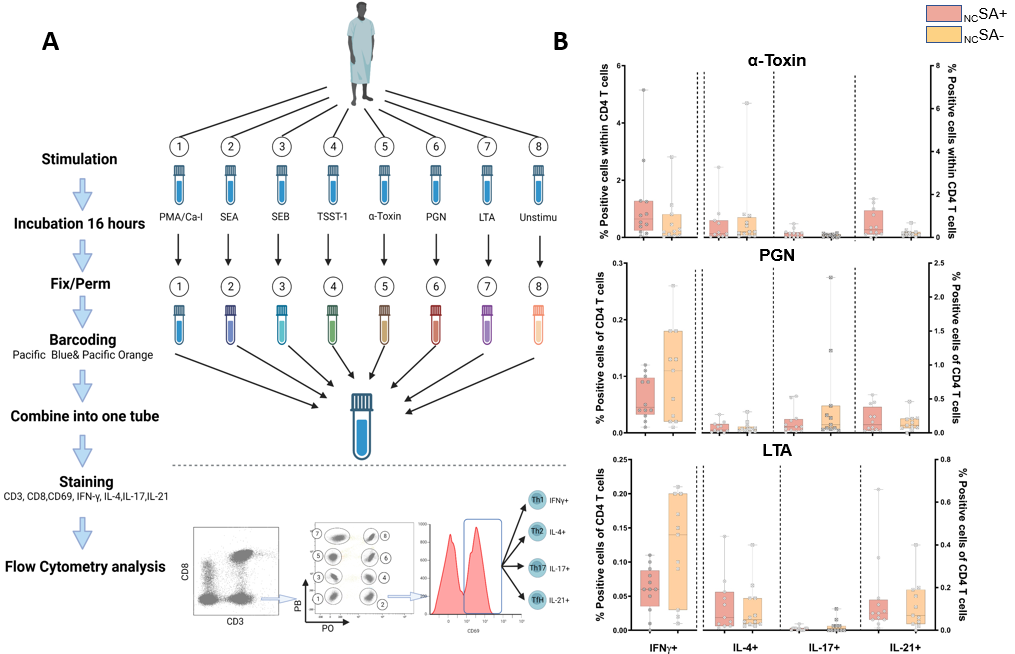


**Supplementary Figure S1**. Overview of the Fluorescent cell barcoding (FCB) method and results for α-Toxin, peptidoglycan (PGN) and lipoteichoic acid (LTA). **(A)** Thawed PBMCs were stimulated with either SEA, SEB, TSST-1, PGN, or LTA. Next, the intracellular cytokine production of IFNγ, IL-4, IL-17, and IL-21 was analyzed in CD69^+^CD4^+^Th cells following stimulation. Details are described in Supplementary Data S2. **(B)** Percentages of IFNγ^+^, IL-4^+^, IL-17^+,^ and IL-21^+^CD4^+^Th cells in the total CD4^+^ T cell population after stimulation with α-toxin, PGN, and LTA in _NC_SA+ (n=12) and _NC_SA- (n=11) remission GPA-patients. Graphs represent Box-and-whiskers plots (Min to Max, Median). _NC_SA+: Non Cotrimoxazole *S.aureus* nasal carrier; _NC_SA-: Non Cotrimoxazole *S. aureus* non-carrier; SEA: staphylococcal enterotoxin A; SEB: Staphylococcal enterotoxin B; TSST-1: Toxic shock syndrome toxin-1; PGN: peptidoglycan; LTA: lipoteichoic acid; PB: Pacific Blue; PO: Pacific Orange. *P*-values were calculated using the nonparametric Mann-Whitney U-test. Partly made with Biorender.com.


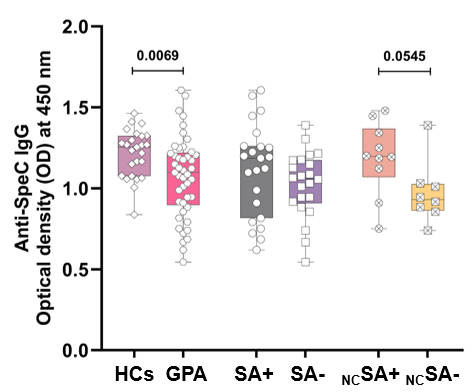


**Supplementary Figure S2. The level of anti-rSpeC IgG in plasma of GPA-patients and healthy controls**. Graphs represent box-and-whiskers plots (Min to Max, Median). rSpeC: recombinant Streptococcal pyrogenic exotoxin C; SA+: *S. aureus* nasal carrier; SA-: *S. aureus* non-carrier; _NC_SA+: Non Cotrimoxazole *SA+*; _NC_SA-: Non Cotrimoxazole *SA-*; HCs: healthy controls.

**
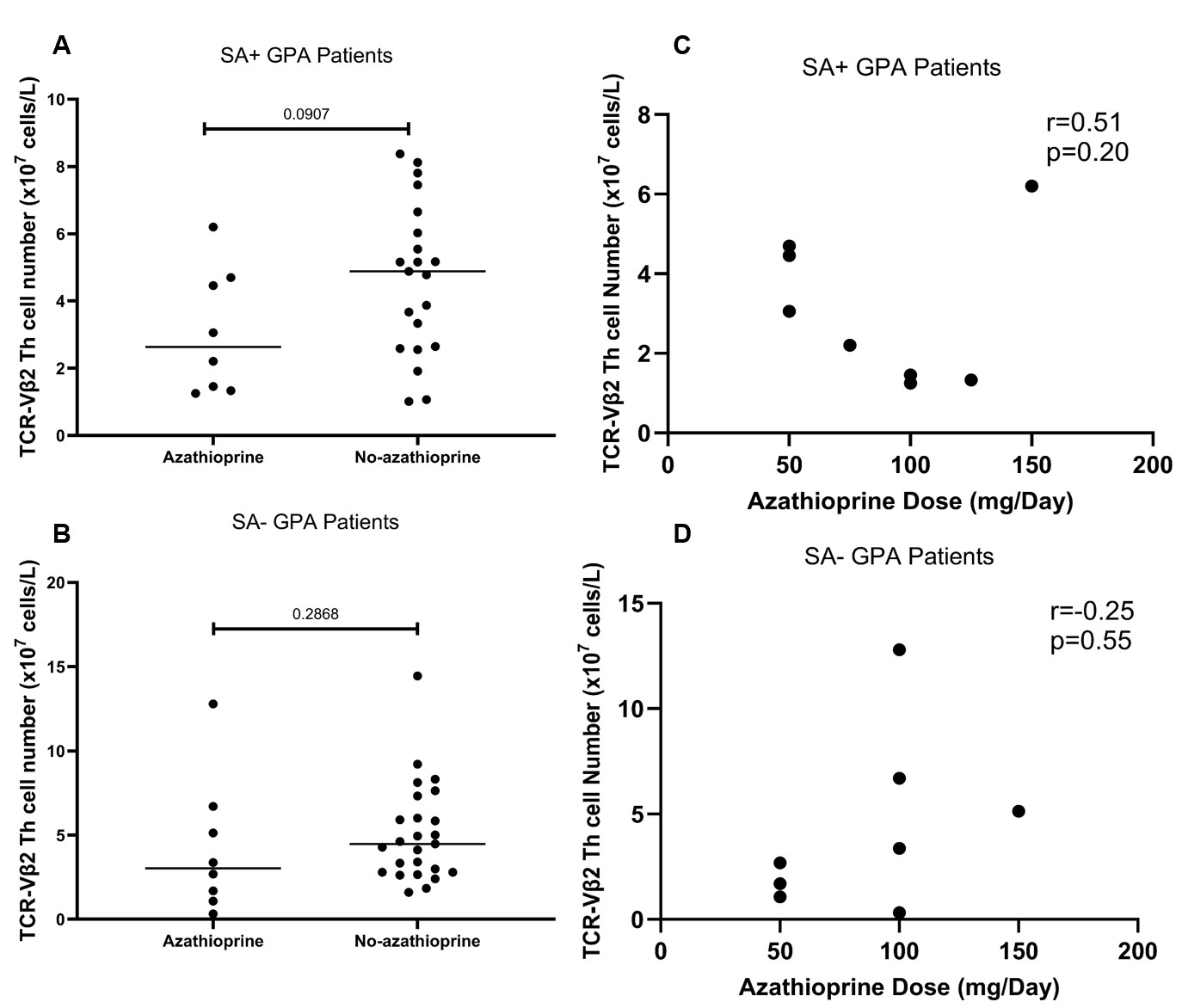
**

**Supplementary Figure S3 Azathioprine effect on the absolute number of TCR-Vβ2^+^Th cells** Graphs show the absolute number of TCR-Vβ2^+^Th cells of patients who received or did not received Azathioprine in SA+**(A)** and SA-**(B)** GPA-patients. Dot plots illustrate the correlation between Azathioprine dose and the absolute number of TCR-Vβ2^+^Th cells within SA+**(C)** and SA-**(D)** GPA-patients. Line represent Median. Non-parametric spearman tests were applied in correlation tests. SA+: *S. aureus* nasal carrier; SA-: *S. aureus* non-carrier.


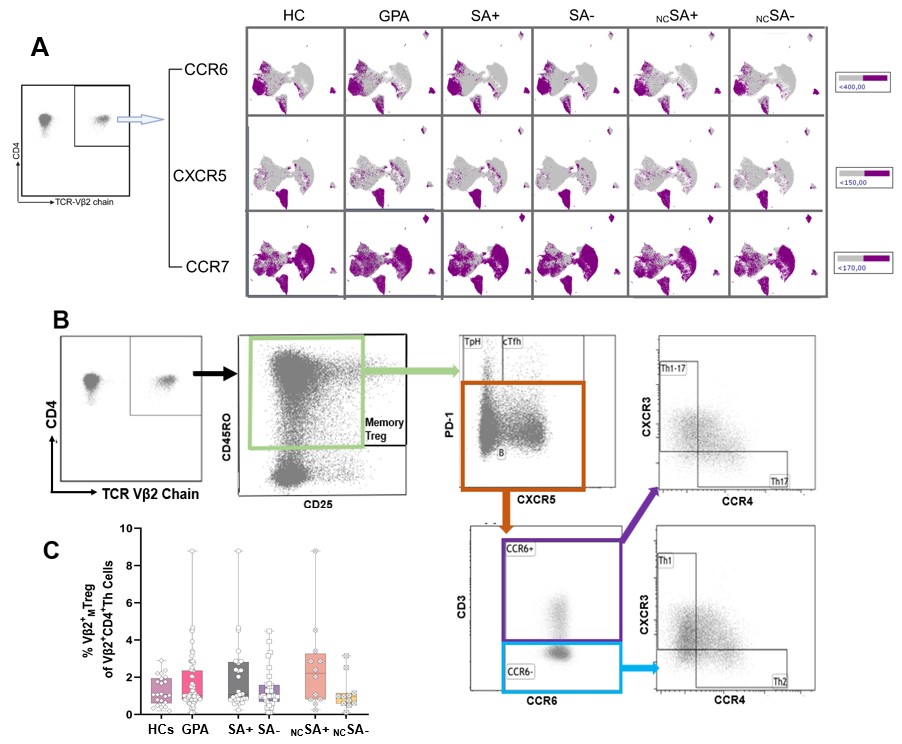
**Supplementary Figure S4**. **UMAPs of surface expression of CCR6, CXCR5, and CCR7 on Vβ2^+^Th cells and manual gating strategy of Vβ2^+^Th cell subsets.** **(A)** UMAP visualization of single marker expression in Vβ2^+^Th cells, generated using 3 × 10^3^ cells from each sample, derived from merged samples of all GPA-patients (n=62) with SA+ (n = 29), SA- (n = 33), _NC_SA+ (n = 12), _NC_SA- (n = 11) and HCs (n = 22). The thresholds were set up based on the values used in manual gating via Kaluza. Numbers represent fluorescence intensity. **(B)** Representative flow cytometry dot plots for identifying circulating Vβ2^+^Th cell subsets in a patient with SA+ GPA in remission. Lymphocytes were first gated based on FSC‐A/SSC‐A properties. After excluding doublets and dead cells, CD4+ T cells were identified as CD3^+^ CD4^+^ lymphocytes. The CD3^+^CD4^+^ T cell population was divided into TCR Vβ2^+^ and TCR Vβ2 ^−^ subsets. The inclusion of CD25 and CD45RO enables further classification of TCR Vβ2^+^ T cells into 7 fractions (38). After fractions 1 and 2 were excluded, TCR Vβ2^+^ Th cells were characterized based on CD45RO expression. T peripheral helper cells (T_P_H) and T follicular helper cells (T_F_h) were identified in the CD45RO^+^ CD4^+^ T helper cell population using PD-1 and CXCR5 expression (T_P_H: PD-1^high^CXCR5^-^; T_F_h: PD-1^high^ CXCR5^+^). Finally, T helper 1 cells (Th_1_, CCR6^-^CCR4^-^CXCR3^+^), T helper 1-17 cell (Th_1-17_, CCR6^+^CCR4^-^CXCR3^+^), T helper 2 cells (Th_2_, CCR6^-^CCR4^+^CXCR3^-^), and T helper 17 cells (Th_17_, CCR6^+^CCR4^+^CXCR3^-^) were delineated based on CCR6, CCR4, and CXCR3 expression in the remaining cells. CCR4 and CXCR3 expression thresholds were based on fluorescence minus one controls (FMOs). **(C)** Percentage of Vβ2+ _M_Treg within memory Vβ2^+^CD4^+^Th cells. Graphs represent box-and-whiskers plots (Min to Max, Median). FSC‐A: forward scatter area; SSC‐A: side scatter area; SA+: *S. aureus* nasal carrier; SA-: *S. aureus* non-carrier; _NC_SA+: Non Cotrimoxazole *SA+*; _NC_SA-: Non Cotrimoxazole *SA-*; HCs: healthy controls. _M_Treg: Memory regulator T cell.

**Supplementary Data S1. Detailed Protocol for Fluorescent Cell Barcoding to Measure Cytokine Production in CD4^+^Th Cells.** Thawed PBMCs were resuspended at 5x10^6^ cells/ml in RPMI1640 (Cambrex Bio Science, Verviers, Belgium) supplemented with 5% FCS and 50 mg/ml gentamycin (Gibco, Scotland, UK). Cell suspensions were aliquoted into 8 polypropylene tubes (5x10^5^ cells per tube) and stimulated with 5ug/ml staphylococcal enterotoxin A (SEA), 5ug/ml Staphylococcal enterotoxin B (SEB), 5ug/ml Toxic shock syndrome toxin-1 (TSST-1), 50ng/ml α-toxin, 1ug/ml peptidoglycan (PGN), or 1ug/ml lipoteichoic acid (LTA) (All Sigma-Aldrich, Zwijndrecht, The Netherlands). To determine the total level of cytokine secretion, one of the cell samples was stimulated with 5ng/ml PMA (Sigma-Aldrich, Zwijndrecht, The Netherlands) and 0,2ug/ml Calcium Ionophore A23187 (Ca-I; Sigma-Aldrich, Zwijndrecht, The Netherlands) as a positive control whereas one cell sample that remained without stimulation was used as a negative control. For optimal co-stimulation, anti-CD28/anti-CD49d (BD Biosciences, Franklin Lakes, USA) was added to each tube at 1mg/ml. After 1 hour, 10 mg/ml brefeldin A (Sigma-Aldrich, Zwijndrecht, The Netherlands) was added to block protein transport. Next, the samples were incubated for 16 h at 37℃ with 5% CO_2_. Following incubation, cells were washed with cold PBS, fixed, and permeabilized in 400 ul of 1X permeabilization buffer (eBioscience, San Diego, USA) containing different concentrations and/or combinations of Pacific Blue (PB, Invitrogen, Carlsbad, USA) and/or Pacific Orange (PO) dyes (Invitrogen, Carlsbad, USA) to enable fluorescent cell barcoding (FCB) in each original tube. Unstimulated samples were stained with 5ug PB+ 10ug PO, whereas samples stimulated with SEA, SEB, TSST-1, α-toxin, PGN, LTA, and PMA+Ca-I were stained with 0ug PB+ 10ug PO, 0.2ug PB+ 0ug PO, 0.2ug PB+ 10ug PO, 1ug PB+ 0ug PO, 5ug PB+ 0ug PO, and 0ug PB+ 0ug PO, respectively. After incubation for 30 minutes at 4℃ in the dark, samples were washed twice with 100% FCS and resuspended in PBS. Next, the different FCB samples were combined into one FACS tube, washed, resuspended in 100 uL of 1X permeabilization buffer, and stained with the following antibodies: anti-human-CD3-APC (BD Biosciences, Franklin Lakes, NJ, USA), CD8-PerCP (BD Biosciences), IL-4-PE-Cy7 (BioLegend, San Diego, CA, USA), IL-17-Alexa Fluor 488 (eBioscience), IL-21-PE (eBioscience), IFNγ-Alexa Fluor 700 (BD Biosciences), and CD69-APC-Cy7 (BD Biosciences). All conjugates were titrated to obtain optimal dilutions and saturating concentrations. After incubation, washing and resuspension, the samples were measured on a BD LSRII Flow cytometer (BD Biosciences, Franklin Lakes, USA). Because stimulation reduces the surface expression of CD4 on T cells, CD4-positive T-cells were identified indirectly by gating CD3-positive cells and CD8-negative lymphocytes. Next, cells from different stimulation tubes were identified based on their FCB signature (as shown in Supplementary Figure S1A), gated separately, and analysed as individual samples for the expression of the activation marker CD69 versus intracellular cytokine production of IFNγ, IL-4, IL-17, and IL-21. Dead cells that were not washed out by several washing steps of the FCB procedure were further excluded from the lived gate according to their forward- and side-scatter patterns. Unstimulated samples were used to set linear gates to distinguish between the positive and negative populations. The results are expressed as the percentage of cytokine-producing CD69+ cells within the total CD4^+^Th cell population. The values were corrected for unstimulated cultures. For all flow cytometry analyses, data were collected for at least 2 x 10^6^ cells and plotted using Kaluza analysis software V2.1 (Beckman Coulter, Indianapolis, USA).

**Supplementary Data S2. Detailed Protocol of the In-House Indirect ELISA for measuring anti-SEA IgG, anti-SEB IgG and anti-SpeC IgG Concentrations.**

For anti-SEA IgG and anti-SEB IgG, briefly, 96-well Nunc MaxiSorp™ flat-bottom immunoplates (Thermo Fisher Scientific, Roskilde, Denmark) pre-coated with purified SEA (0.2ug/well, Sigma-Aldrich, Zwijndrecht, The Netherlands) or purified SEB (0.2ug/well, Sigma-Aldrich, Zwijndrecht, The Netherlands) were incubated (16 h at 25°C), followed by washing (0.05 % Tween-20 in PBS)-and blocking (2 % BSA in PBS; 1 h at 25°C) steps. Plasma and secondary antibody (mouse anti-human-IgG–HRP, SouthernBiotech, Birmingham, USA) were diluted (1% BSA in PBS), and incubated for 1 h at 25°C. The reaction was initiated with TMB substrate (Sigma-Aldrich, St Louis, USA) and was stopped with sulfuric acid. Optical density was measured using a VersaMax ELISA Microplate Reader (Molecular Devices, San Jose, USA) at a wavelength of 450nM and processed using SoftMax Pro 7 software v7.0.3 (Molecular Devices, San Jose, USA).

For anti-SpeC IgG, briefly, 96-well Nunc MaxiSorp™ flat-bottom immunoplates (Thermo Fisher Scientific, Roskilde, Denmark) pre-coated with purified rSpeC (Tox Tech, Sarasota, USA) were incubated (16 h at 4°C), followed by washing (0.8g/L Tween-20 in PBS)-and blocking (10g/L BSA in PBS; 2 h at 25°C) steps. Plasma samples were diluted (1g/L BSA in PBS) and incubated for 16 h at 4°C. Secondary antibody (mouse anti-human-IgG–HRP, SouthernBiotech, Birmingham, USA) were then diluted (1g/L BSA in PBS) and incubated for 2h at 25°C. The reaction was initiated with TMB substrate (Sigma-Aldrich, St Louis, USA) and was stopped with sulfuric acid. Optical density was measured using a VersaMax ELISA Microplate Reader (Molecular Devices, San Jose, USA) at a wavelength of 450nM and processed using SoftMax Pro 7 software v7.0.3 (Molecular Devices, San Jose, USA).

**Supplementary Data S3. Detailed Protocol for the In-House Sandwich ELISA to quantify Total IgG Concentration.** In brief, 96-well Nunc MaxiSorp™ flat-bottom immunoplates (Thermo Fisher Scientific, Roskilde, Denmark) were coated with 1.3 μg/ml AffiniPure F(ab')₂ Fragment Goat Anti-Human IgG (Jackson ImmunoResearch Laboratories Inc., West Grove, USA). After blocking, diluted supernatant was added to the coated wells. As standard-curve purified human IgG was used (Siemens, Marburg, Germany). Next, Mouse Anti-Human IgG Fc-HRP Antibody (SouthernBiotech, Birmingham, MA, USA) was used as the secondary antibody. Then, the samples were incubated with TMB substrate (Sigma-Aldrich, St Louis, USA) and the reaction was stopped with sulfuric acid. Optical density was measured using a VersaMax ELISA Microplate Reader (Molecular Devices, San Jose, USA) at a wavelength of 450nM and processed using SoftMax Pro 7 software v7.0.3 (Molecular Devices, San Jose, USA).
